# Optically Tunable Magnetoresistance Effect: From Mechanism to Novel Device Application

**DOI:** 10.3390/ma11010047

**Published:** 2017-12-28

**Authors:** Pan Liu, Xiaoyang Lin, Yong Xu, Boyu Zhang, Zhizhong Si, Kaihua Cao, Jiaqi Wei, Weisheng Zhao

**Affiliations:** 1 Fert Beijing Research Institute, School of Electrical and Information Engineering, Big Data and Brain Computing Center (BDBC), Beihang University, Beijing 100191, China; liupan@buaa.edu.cn (P.L.); yong.xu@univ-lorraine.fr (Y.X.); boyu.zhang@buaa.edu.cn (B.Z.); szz931220@buaa.edu.cn (Z.S.); kaihua.cao@buaa.edu.cn (K.C.); weijiaqi@buaa.edu.cn (J.W.); 2 Beihang-Geortek Joint Microelectronics Institute, Qingdao Research Institute, Beihang University, Qingdao 266000, China; 3 Institut Jean Lamour, CNRS UMR 7198, Université de Lorraine, 54506 Vandœuvre-lès-Nancy, France

**Keywords:** spintronics, magnetoresistance effect, optically tunable, interlayer exchange coupling, data storage

## Abstract

The magnetoresistance effect in sandwiched structure describes the appreciable magnetoresistance effect of a device with a stacking of two ferromagnetic layers separated by a non-magnetic layer (i.e., a sandwiched structure). The development of this effect has led to the revolution of memory applications during the past decades. In this review, we revisited the magnetoresistance effect and the interlayer exchange coupling (IEC) effect in magnetic sandwiched structures with a spacer layer of non-magnetic metal, semiconductor or organic thin film. We then discussed the optical modulation of this effect via different methods. Finally, we discuss various applications of these effects and present a perspective to realize ultralow-power, high-speed data writing and inter-chip connection based on this tunable magnetoresistance effect.

## 1. Introduction

**Figure 1:**
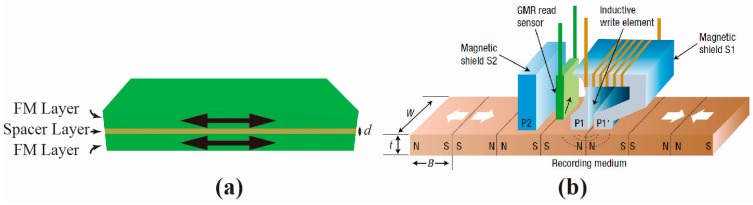
(**a**) Schematic of a GMR device with a FM layer/spacer layer/FM layer stacking. The thickness of the spacer layer is labeled as d; (**b**) GMR read-head for hard drive [2]. Reproduced with permission from [2].

The appreciable magnetoresistance (MR) effect known as giant magnetoresistance (GMR) and tunneling magnetoresistance (TMR) appears in an artificial, nano-scale, sandwiched structure consisting of two ferromagnetic (FM) layers separated by a nonmagnetic spacer (Figure 1a). Its successful application has completely revolutionized the information industry and changed our daily life [1,2,3]. The underlying process of these MR effects is a switch of the relative magnetization arrangement, between an antiparallel (AP) arrangement and parallel (P) arrangement, during a sweep of the magnetic field. Such a magnetization switch induces a large change in electrical resistivity of the multilayers. The change is usually several orders of magnitude larger than the anisotropic magnetoresistance (AMR) effect [4]. The discovery of these appreciable MR effects has paved a way for transforming weak magnetic information into a large electrical signal, leading to numerous impactful applications. Representatively, the GMR- and TMR-reading head in a hard-disk drive (HDD) has boosted the computer storage density and capacity, which promoted the era of big data (Figure 1b) [2,5]. Additional design and optimization of the GMR/TMR sensors have further enabled applications of position and/or speed sensing [6,7], and even biological probing [8]. Moreover, the emerging nonvolatile magnetic memory (MRAM) based on the TMR effect has been widely considered as a competitive choice for next generation universal memory [2]. The integration of these nonvolatile memory devices with complementary metal oxide semiconductor (CMOS) devices has been successfully applied for high-performance logic circuits [9]. Besides these direct applications of the GMR/TMR effect on sensing, data storage and processing, breakthroughs in the optical tuning of these effects further promise ultra-fast, high-volume data transmission solutions, which meet the ever-growing speed and bandwidth demands of inter-chip communication.

In the following sections, firstly we review the GMR/TMR effect and the IEC effect in different material systems, concurrently we discuss the related basic and forefront key research issues. Then we revisit the development and principles of the optical manipulation of the MR effect. Such manipulation can be realized via different methods, including switching the relative magnetic alignment via the all optical switching (AOS) of the magnetic layer or via tuning the IEC effect; or otherwise modulating the electronic transport of the optical responsive spacer layer. The final section is devoted to the abundant applications of those effects, including data storage and sensing. At the end we present a perspective for applying the optical tunable MR to realize ultra-low-power optical date writing and high-speed, inter-chip connection.

## 2. Magnetoresistance and Interlayer Exchange Coupling Effect

### 2.1. GMR/TMR Effect

The GMR and TMR effects originally describe the appreciable resistance change, during a sweep of applied magnetic field, of a magnetic sandwiched structure with a spacer layer of nonmagnetic metal or insulator, respectively. Here the magnetic field serves as a tool to switch the relative magnetization of magnetic layers. The system would exhibit relatively high resistance in antiparallel alignment, with low resistance in the parallel alignment.

In 1988, the GMR effect was first discovered in antiferromagnetic (AFM) interlayer-exchange-coupled Fe/Cr multilayers by two research groups separately led by A. Fert and P. Grünberg [10,11]. The AFM IEC, which we will discuss later, guarantees that the adjacent layers will be at AP alignment in its natural state. This makes it possible for the applied magnetic field to force a contrastive P alignment [5]. However, these coupled systems show low sensitivity to the magnetic field due to an enhanced saturation field, which can be a huge flaw for practical applications. Researchers then developed non-exchange coupled structures in which two magnetic layers have different cohesive fields [12,13], called pseudo spin-valves, or where one of them is magnetically pinned by an additional pinning layer via the exchange bias effect, called a spin-valve [14,15]. The spin-valve structure has greatly contributed to the successful application of GMR [5]. Apart from different approaches for creating distinct magnetic alignments, a GMR device also has different device geometries. At the beginning, GMR was detected by electric current flowing in the film plane (CIP), and later research developed the “current perpendicular to plane (CPP)” geometry [16,17,18] that shows a relatively stronger effect [19]. The GMR effect rapidly found impactful applications in data storage, and attracted tremendous research interest.

The success of the GMR effect strongly incentivized research into the TMR effect. The very first report on TMR actually came before the discovery of GMR [20]. However, that experiment had been performed at low temperature and was hardly reproducible. The breakthrough of appreciable and reproducible TMR at room temperature (RT) was achieved when amorphous AlO_x_ was adopted as a tunneling barrier in the magnetic tunnel junction (MTJ) [21,22]. TMR as large as 81% at RT has been achieved in an optimized AlO_x_–MTJ system [23]. With novel applications like MRAM still craving higher MR ratios, researchers continued exploring different materials and found by calculation [24], and then revealed by experiments, that MgO as barrier material could provide up to 1000% TMR ratio at 5 K [25,26,27]. MgO/CoFeB-based MTJ with perpendicular magnetic anisotropy (PMA), which presents higher switching energy efficiency and extra scalability, has become the mainstream for MRAM applications [28,29].

Along with the development of the magnetoresistance effect, the underlying physical mechanism has been better understood. The microscopic mechanism of the GMR and TMR effects is about electron transport dominated by spin-dependent scattering [30,31] or tunneling [20,24] respectively. As described by Mott’s two-current model [32,33], electrons of spin-up or spin-down can be imagined to transmit through two independent channels. If the multilayer is in parallel arrangement, electrons with a spin direction the same as the magnetization direction will be less scattered (for GMR) or have higher tunneling probability (for TMR), resulting in low resistance. Conversely, electrons with a spin direction opposite to the magnetization direction will encounter a large resistance. Thus, the total resistance of the two channels in parallel connection will be relatively small, while if the multilayer is in an antiparallel arrangement, both channels will encounter a large resistance, hence leading to a large total resistance [2]. To realize such a spin-dependent interaction, electrons, which convey the spin information, should be able to maintain their spin momentum. This is why materials of spacer layer with a long mean-free-path (for CIP GMR), spin-diffusion-length (for CPP GMR) and small thickness (for both GMR and TMR) are necessary for an appreciable GMR/TMR effect. Values of these MR effects will depend on the spin-polarization of magnetic materials, the maintenance of spin momentum across spacer/barrier materials and the interfaces, and additional spin filtering effects induced by specific tunneling barriers [34]. As a result, well controls of the ferromagnetic materials, spacer/barrier materials and their interfaces, together with the emergent techniques of magnetic switching and MR effect modulation have been considered as key issues of spintronic research [1,35,36].

### 2.2. Interlayer Exchange Coupling Effect

The discovery and development of the GMR effect, which relies on the manipulation of the relative magnetic arrangement, has been intimately linked to the research achievement of the IEC effect [37,38,39]. IEC describes the magnetic interaction between two FM layers, mediated by a nanometer-thick spacer layer. IEC can be AFM or FM, depending on the spacer layer with specific thickness, which determines whether AP or P is the energy favorable state [40]. The IEC has been systematically established in numerous layered structures with different metallic spacer layers [41,42,43,44], as well as some semiconducting, insulating and organic molecular spacers e.g., Si [45,46,47], GaAs [48,49], MgO [50,51] and α-sexithiophene [52]. More interestingly, IEC strength is found to oscillate periodically between AFM and FM states, with varying thicknesses of metallic spacer layer [41,42,43,53].

The unusual phenomena of IEC has attracted a great deal of research interest in its mechanism. Microscopically, IEC is an indirect exchange interaction mediated by the electrons of the spacer layer. Pioneering theoretical researchers have tried to develop a unified theory for both metallic and insulating spacer layers, by introducing the concept of a complex Fermi surface [54,55]. A more recent and commonly accepted theory is that the oscillatory IEC is mediated by the quantum well states (QWS), which have been experimentally observed to occur in the spacer layer [40,56]. QWS describes the discrete electronic states formed by electron confinement, and those states evolve periodically with the well width [40,57,58]. The theoretical explanation of the IEC effect by QWS can be briefly interpreted as follows.

The IEC coupling strength *J* (value positive for AFM and negative for FM coupling, indicating the energy minimization principle) is determined by the energy difference between AFM and FM coupling states (*E_FM_* and *E_AFM_* represent the energy of the FM and FM coupling states, respectively) [40]:

(1) $$2J=E_{FM}-E_{AFM}$$

**Figure 2:**
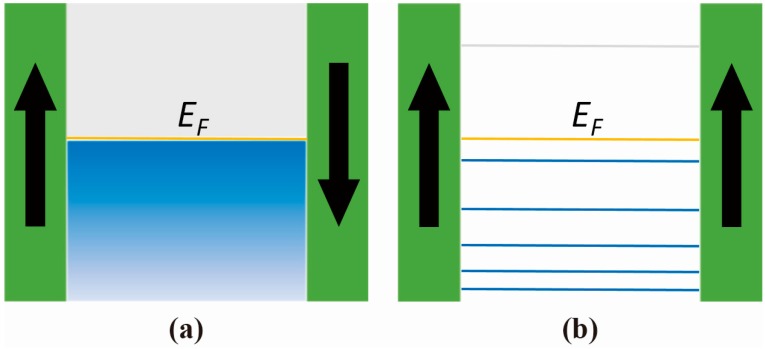
Electron distribution schema of the (**a**) AFM coupling and (**b**) FM coupling state. At the ground state, electrons occupy only those states below the Fermi level (*E_F_*) (occupied states are colored blue, unoccupied colored gray).

The energy of electron gas in the spacer layer can be obtained by

(2) $$E=\int_{-\infty }^{+\infty }\varepsilon D(\varepsilon )f(\varepsilon )d\varepsilon$$

where *D*(*ε*) represents the density of states (DOS), and *f*(*ε*) represents the distribution function [40,55]. Due to the splitting of the bands in the magnetic materials, the electrons in the spacer layer with their spins opposite to the magnetization are strongly reflected at the interfaces between the FM layer and the spacer layer. Thus, when two magnetic layers are aligned parallel (direction ‘up’ in our example), as illustrated in Figure 2b, spin down electrons are strongly reflected at both interfaces, like being trapped in a well, which leads to their confinement. In contrast, such a quantum well situation cannot occur when the two magnetic layers are aligned antiparallel, as in Figure 2a, because either spin up or spin down electrons can always penetrate one of the two interfaces with little reflection. Therefore, the DOS of spin up electrons in the FM coupling state (*D_FM_*) is strongly altered by confinement into nearly a set of delta functions at discrete energy levels, different from the continuous DOS of the AFM coupling state (*D_AFM_*), as shown in Figure 2a,b. According to Equation (2), these different coupling states result in an inequity between *E_FM_* and *E_AFM_*. Such an energy difference ultimately determines the natural preference of one magnetization arrangement over another, which is manifested as the different types of IEC. Following this theory, the oscillatory behavior of IEC in certain material systems can be well understood. As the thickness of the spacer layer increases, the QWS energy levels shift downwards according to quantum mechanics, which generally decreases the *E_FM_*. However, when a QW state crosses the Fermi level (*E_F_*) from above, it adds to the integration term in Equation (2), meaning that *E_FM_* increases sharply, which is when FM coupling turns out to be unfavorable [40]. Therefore, changes of the *E_AFM_* contribute to the alteration of two types of IEC. (More details of this mechanism can be found in Reference [5]).

Based on the mechanism of the IEC effect, it is possible to design magnetically-coupled multilayers with negligible influence to other adjacent layers (that is, synthetic anti-ferromagnet (SyAF), see references [36,59,60]). The SyAF technique has already contributed to the booming of the HDD market and the development of MRAM. Besides, the IEC effect also implies a tricky strategy to realize magnetic switching by switching the coupling type [52,61,62,63,64,65].

### 2.3. MR in Different Material System

Traditional MR structures usually consist of two FM metal layers separated by a nonmagnetic metal spacer (for GMR) or a metal-oxide barrier (for TMR). As indicated by the mechanism of the GMR/TMR effect, the choice of the spacer can affect the GMR/TMR effect by various critical aspects, including interface lattice match, spin polarization, spin-diffusion length, mean-free path, etc. The observation of GMR first succeeded in the molecular beam epitaxy (MBE)-grown, Fe/Cr/Fe, AFM-coupled, nanometer-thick multilayers with well-defined interfaces [10,11], and later systematically extended to various nonmagnetic spacer-based systems, including Fe/Cr, Co/Ru, etc. [41,42,43,44]. Among them Co/Cu, whose well-matched crystal structures minimize interface defects and thus consequential spin-independent scattering, showing a strong MR and IEC effect, making it an archetypical GMR system [1,42,44]. As for TMR, despite some early observations regarding the Ge-based junction [20], strong TMR at RT was not achieved until the amorphous AlO_x_ barrier was adopted [21,22]. A further milestone has been the theoretical prediction and successful observation of a high TMR ratio in MgO-based systems, where the selective tunneling property of MgO with certain crystal orientations enhances spin polarization [24,25,26]. With the MgO-based junction prevailing in current TMR applications for its large TMR ratio, researchers continue searching for novel material systems [66,67,68], including the choice of capping materials [69,70,71], towards better device performance, such as larger TMR, a lower resistance area (RA), higher breakdown voltage and lower spin-torque switch current density [28,29].

Apart from traditional MR structures, tempted by relatively long spin diffusion length and the potential of electrical and optical modulation methods in semiconductors [1,72], plentiful attempts have been made to develop MR devices based on semiconductor spacers, including AlAs, GaAs, Si etc. [72,73,74]. Unlike all-metal systems, several key issues still remain to be tackled for semiconductor spacer-based hererostructures. These issues include the lattice mismatch and intermixing effect which result in poor interfaces between the FM metal and semiconductor, together with the impedance mismatch that makes the MR too weak to be detected [72,75,76]. Possessing the same advantage of long spin diffusion length as semiconductors, organic materials furthermore promise economic mass fabrication and flexible property manipulability from the material’s perspective [77,78,79,80]. Various types of organic materials have been employed as a spacer material, with moderate MR ratios achieved, notably carbon nanotubes [81,82] and small organic molecules like sexithienyl (T_6_) and 8-hydroxy-quinoline aluminium (Alq_3_), etc. [83,84,85,86]. Additionally, the photosensitivity of certain organic materials allows the combination of the MR effect and photoresponse in a single device, as researchers recently proved using fluorinated copper phthalocyanine (F_16_CuPc) and C_60_ fullerene spacer-based devices [87,88]. A similar case lies in the poly(vinylidene fluoride) (PVDF)-based MTJ, which enables both magnetic and ferroelectric control of the device [89,90]. Besides exploring spacer or barrier materials, attempts have also been made to apply novel magnetic layer materials, for instance half-metals, which provide fully polarized electrons [91,92], or defect-induced magnetism (DIM) material [93,94], or materials with magnetization tunable by light [95,96,97], voltage [98,99], heat [100], etc. The introduction of these novel MR material systems, which either provide an improved MR ratio or permit additional methods of tuning MR, greatly adds to the theoretical and applicational richness of the MR effects.

## 3. Optically Tunable MR Effect 

Since the MR effect predominantly relies on the relative arrangement of the two magnetic layers, a key issue of its application is the ability to control the relative magnetic orientation [101]. Several approaches have been put forward: (1) The very intuitive magnetic field switching approach; (2) The electric current switching approaches using spin transfer torque (STT) [102,103,104] or spin orbit torque (SOT) mechanisms [105,106,107]; (3) Novel electric field, heat or strain-assisted approaches [108,109,110,111]. However, the operating speed of those approaches are ultimately constrained by the spin precession time [112]. Moreover, those electric approaches always have bottlenecks of bandwidth and data loss issues for high-speed, inter-chip communication. Given that, for highly demanding novel device applications, optical approaches for high-speed MR modulation or even magnetic switching have been pushed to the frontier of research [35]. The realization of optically tunable magnetoresistance (OTMR) promises the integration of the ultra-fast, high-volume feature of optical information transmission with the non-volatility, high-density features of spintronics magnetic storage.

**Figure 3:**
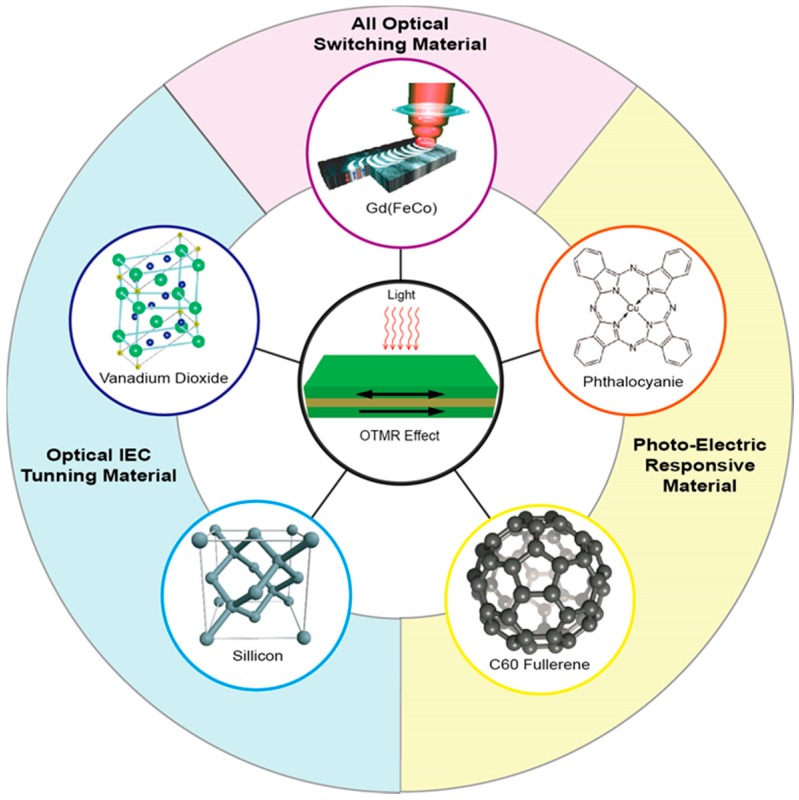
Examples of potential materials for the OTMR effect. Including AOS materials for the FM layer [95]; and phase-transition material VO_2_, organic and inorganic photosensitive materials for the spacer. Reproduced with permission from [95].

Intuitively there are different viable solutions for the optical approach: light can be applied either to the magnetic layer for a direct influence on magnetization [95,96,97,112]; or to the spacer layer, to tune the IEC, which would consequently affect the magnetic arrangement [61,64,65], or otherwise to tune the electric transport properties [87,88,113,114]. The material choice for the former could be the AOS magnetic materials, whose magnetization can be switched directly by light. The latter demands materials with electronic properties effectively tunable by light, such as VO_2_ with a metal-insulator transition (MIT) feature, optically-sensitive semiconductor, and Phthalocyanie (Pc), etc. (Figure 3).

### 3.1. All Optical Switching

Motivated by the demands of high-speed, large-volume storage applications, researchers have exploited using light instead of magnetic fields to manipulate magnetization in data recording materials. While laser has already been used as a heating source in the so-called heat-assisted magnetic recording (HAMR) to assist magnetic field-driven switching [115], early theoretical and experimental studies meanwhile confirmed light as an electro-magnetic wave can directly influence the magnetization [116]. Major breakthroughs of AOS, which means deterministic magnetic switching triggered purely by femtosecond laser pulse, have been achieved during the last decade. Two types of AOS have been observed experimentally, namely all optical helicity-dependent switching (AO-HDS) and all optical helicity-independent switching (AO-HIS), depending on whether the magnetic switching relies on the helicity of light. AO-HDS was once believed to be an effect limited to the rare earth-transition metal alloy [95]. More recently, general principles for designing and fabricating AOS material systems have been put forward, broadening the AOS material choices to include synthetic ferrimagnetic multilayers and heterostructures, as well as RE-free pure ferromagnetic [Pt/Co], [Ni/Co] multilayers, etc. [96,117], transparent medium Cobalt-substituted yttrium iron garnet (YIG:Co) [118], and high-anisotropy FePt film which is a commonly used HAMR media [117]. The other type of AOS, the AO-HIS, has been discovered in GdFeCo alloy [119]. The magnetization of GdFeCo switches after each single pulse of femtosecond laser independent of the light helicity. The switching process is driven by the ultrafast heating with a signature of transient ferromagnetic states [120]. Apart from this direct switching by light, recent studies also found another switching mechanism for GdFeCo capped by thick metal layers, which contributes the switching to indirect hot electrons generated by light and propagating through the metal layers [121,122]. Despite intensive theoretical investigation dedicated to this, ambiguity still shadows the mechanism of the AO-HDS. Many fundamental questions remain to be answered, such as the role of the domain size [123], the role of optical spin transfer torque [124], the contribution of magnetic circular dichroism [125] and the role of the inverse Faraday effect [126].

**Figure 4:**
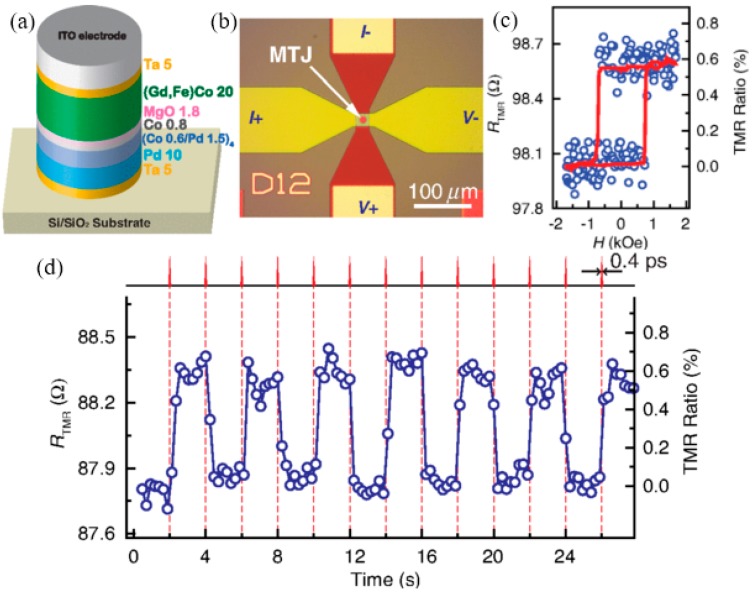
Device demonstration of AOS in an MTJ with subpicosecond single laser pulses without external magnetic field at RT. (**a**) Schematic of the MTJ structure used in the experiment; (**b**) Optical microscope image of a typical MTJ device with an indium tin oxide (ITO) electrode on the top for TMR measurement; (**c**) The *R*_TMR_(H) minor loop measured by sweeping a perpendicular magnetic field, which switches the Co/Pd layers (*R* and *H* represent resistance and magnetic field separately). The red line is the smoothing of the raw data (open circles); (**d**) *R*_TMR_ of the MTJ device measured during AOS by 0.4-ps single laser pulses at 0.5-Hz repetition rate. The changes of *R*_TMR_ in (**c**,**d**) have the same value of ~0.6 ± 0.05 Ω, indicating the GdFeCo layer has been completely switched. Reproduced with permission from [112].

Although the research into AOS is still in its early stage, people have already been attempting to bring it into application. Recently a pioneering demonstration of a GdFeCo-based AOS MTJ device was accomplished. Its free layer and pinned layer materials are GdFeCo and Co/Pd, respectively. The switching between parallel and antiparallel configurations was achieved by switching the GdFeCo using femtosecond laser pulse, although only a low MR ratio of 0.6% was achieved at RT (Figure 4) [112]. Moreover, in another newly reported experiment, a picosecond electric pulse of 9 ps was optically generated by a photoconductive switch. Such a picosecond electric pulse can induce ultrafast magnetic switching in GdFeCo toggles, which implies possible applications for ultrafast spintronic devices [127]. Further progress of this field will strongly rely on the development of on-chip photonics, emerging materials with lower requirement of the laser, and advanced device applications.

### 3.2. Optical Tuning of IEC Effect

Manipulations of the GMR/TMR effect usually depend on the direct switching of magnetization in the FM layer, for example, via a magnetic field, a spin current or a laser pulse. However, the existence of coupling between neighboring FM layers implies the possibility of realizing the switching via a control of the IEC type. Since the IEC effect relies on the electronic properties of the spacer layer, in theory it could be effectively tuned by light in devices based on an optically sensitive spacer. Following the underlying QWS mechanism of IEC as stated earlier, we can understand from the energy’s perspective the consequence that if the spacer layer is exposed to certain photon irradiation with sufficient fluence, owing to the different DOS, the electron gas in the AFM and FM coupling states will separately go through different absorption-transition behaviors, thus bringing about different light-induced energy changes. This could consequently change the relative magnitude between *E_AFM_* and *E_FM_*, which would induce switching between two states as a consequence.

Some experimental and theoretical studies have revealed its feasibility. Pioneering work has been carried out with semiconducting spacer-based systems. In 1993, a photon-induced IEC change from FM coupling to AFM coupling in Fe/(Fe-Si) superlattices at low temperature was report [61,128], though with certain controversy [62,64]. Apart from optically-sensitive semiconductors, another notable material proposal is the VO_2_, which features the MIT property [65]. Researchers performed first principle calculations of the IEC effect between Co-doped, TiO_2_/VO_2_-diluted magnetic semiconductor multilayers. Their results indicated that reversible switching from FM IEC to AFM IEC can be realized utilizing the temperature-induced MIT feature [65], which might be induced by light as well. Thanks to this progress in different material systems, device demonstration via optically-tuned IEC may be realized in the near future.

### 3.3. Optically Sensitive MR Effect

In some material systems, unlike the previous two cases, light illumination on the spacer do not necessarily provoke deterministic switching of the magnetic alignment, while the MR can still be effectively tuned due to the alteration of electronic transport properties [87,88,113,114]. Representatively, such effects are achieved in fluorinated copper phthalocyanine (F_16_CuPc) and C_60_ fullerene-based spin valve structures [87,88]. In the former system, photo-generated charge carriers in the spacer dominate the electric conductivity of the system [87]. Yet in the later, photon irradiation generates a photovoltage [88]. In both cases, the MR effect can be superimposed on the photoresponsive effects. Therefore, by cooperatively adjusting the light irradiation and the applied magnetic field, we can either obtain controllable multiple resistance states, or eliminate the base current of the MR effect [87,88], which can have abundant applications in high-density data storage and neuromorphic devices [129].

## 4. Application

### 4.1. Application of the GMR/TMR Effect

The rapid adoption of the GMR/TMR HDD head has long been regarded as a successful example of fundamental research advances quickly transforming into significant commercial applications. Thanks to the introduction of the GMR/TMR HDD head, we witnessed the capacity of HDD to grow by over thousands of times in two decades, intriguing a revolution in data-storage which constitutes the basis of this information era [2]. Besides, possessing unique high sensitivity and large response in such a small size, the GMR/TMR magnetic sensor can be specifically designed to fit in a vast range of application scenarios. For example, scalable down to sub-μm size, the TMR sensor permits very high spatial resolution, making it suitable for high-precision position, angle and motion sensing [19,130,131,132]. Also, the MR sensors are sensitive enough for detecting geomagnetic fields; meanwhile they can be integrated into integrated circuit (IC) chips, which makes them widely adopted for navigation, posture detection, etc. [19,130,133]. With the boom of the Internet of things (IoT), these sensing applications are becoming ubiquitous, from daily life to industry management. Another promising field for GMR/TMR sensors consists in biosensing, where they are used to detect the surface binding reaction of certain biological molecules labeled with magnetic particles, enabling non-invasive, quick and inexpensive medical diagnosis [130,134,135,136]. Still, MR sensors can find broader niche applications, like detecting defect regions in metal parts, monitoring current density in IC chips, etc. [130,133,137].

Moreover, as the pillar of spintronics, the MR effect has the potential to play a major role in the beyond-Moore era [35]. One great dilemma of today’s electronic industry is the ever-increasing power consumption brought on by growing computing demands, contradictory to the pursuit of portability and the compaction of products. TMR-based MRAM, which features certain key advantages including non-volatility, low-voltage operation, high-speed, and nearly infinite endurance, permits alleviating this issue [36,138]. Major chip fabricators have been targeting MRAM as embedded memory to substitute current volatile RAMs. With its scalability, MRAM also has the potential to be applied to large volume data storage [139,140]. Besides, a novel conceptional magnetic data-storage device named racetrack memory, which uses the TMR effect to read information stored in dynamic magnetic domains or skyrmions, is also under development [141,142].

Lastly, the development of GMR/TMR effects also benefits spintronics-based logic applications. The integration of MTJ-based memory with CMOS has been successfully applied for high-performance logic circuits [9]. Moreover, the development of GMR/TMR has boosted research advancements into magnetic and barrier materials, together with the control of the interfaces. Those achievements continue promoting various spintronics-based logic applications such as all-spin-logic, spin wave logic, etc., which highly rely on efficient spin-charge conversion and the modulation of spin propagation [143,144].

### 4.2. Application of OTMR Effect

The light-tunable MR effect indicates a novel path for combining photonics with magnetic technologies [112]. The first intriguing application consists of the data writing of magnetic memory. The optical writing of a novel AOS-material-based memory bit can be achieved with a single femtosecond laser pulse [95,121], requiring the switching energy prospectively to be much lower than the current electrical switching approaches [145]. Admittedly, before reaching a point of practical application, it still demands further research for AOS material systems with higher MR ratios, and efforts at the device engineering level to realize downscaled devices switchable by low-power laser. Different to the optically-switchable MR devices, which mainly provide higher speed and power-efficiency, the optically-sensitive MR devices feature other advantages. For example, stable multiple resistance states can be achieved in a single optically-sensitive MR device, which permits improved density for data storage applications, or otherwise can find its place in various novel neuromorphic applications. On the other hand, an optically-sensitive, zero-base-current MR device can function with significantly lower power-consumption [88].

**Figure 5:**
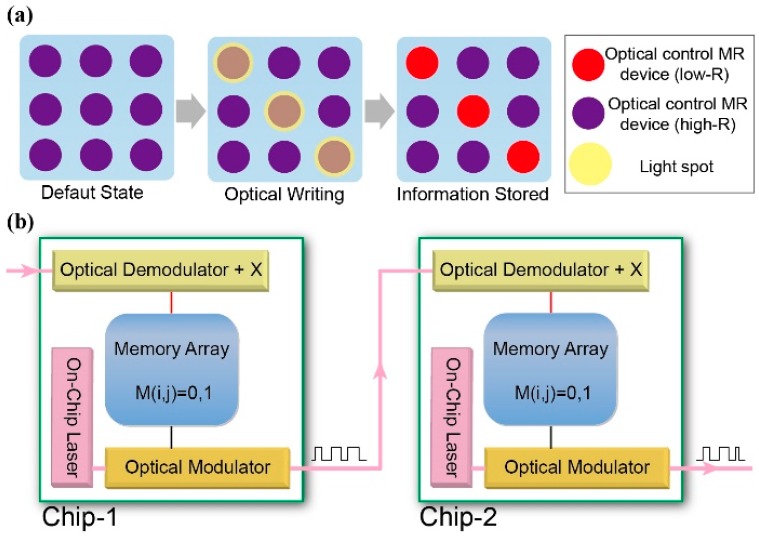
Schematic of the potential applications of the OTMR effect (**a**) Data writing in optical control MR chip. This chip, based on the OTMR devices array, can serve as a memory module in the following chips in (**b**); (**b**) Inter-chip optical communication. According to the data stored in the “Memory Array” on Chip-1, the laser beam from the “On-Chip Laser” can be modulated by the “Optical Modulator” to convey the information. Once another chip (“Chip-2”) receives the modulated laser beam from Chip-1, the “Optical Demodulator + X” unit will demodulate the beam and then write the “Memory Array” on Chip-2 in an optical writing way utilizing the laser-induced change of MR (the “X” may be a laser demultiplexer to perform selected data writing into specific memory unit).

As a perspective, once the on-chip laser technology matures, and a breakthrough of the high-MR AOS material systems arises, the optically-switchable MR will enable the integration of the ultra-fast, high-volume optic information transmission technology and the non-volatile, high-density spintronics magnetic storage technology, which would inaugurate a new vision of efficient data writing and inter-chip communication (Figure 5).

## 5. Conclusions

The collision and blending of magnetics, electronics and nanotechnology have triggered the birth of spintronics, which is marked by the discovery of the GMR and TMR effect. These magnetoresistance effects and other emerging effects, with abundant applications in the information industry, have kept changing our daily life for several decades. In this paper, we have reviewed the development of GMR, TMR and other related effects, from their mechanism to novel device applications. We first revisited the discovery and mechanism of GMR, TMR and IEC effects within various material systems. We then reviewed the optically tunable MR effect by different approaches. Finally, we discussed the abundant applications of these MR effects and presented a perspective to realize efficient data writing and inter-chip communication.
